# Cardiac arrest associated with non-toxigenic *corynebacterium diphtheria* strain: A case report

**DOI:** 10.3389/fmed.2022.1003193

**Published:** 2023-01-09

**Authors:** George V. Alvarez, Deborah D. Rupert, Samantha Sinclair, Santiago J. Miyara, Sara Guevara, Peter Shih, Cynthia Hoey

**Affiliations:** ^1^Department of Family Medicine, Glen Cove Hospital, Northwell Health, Glen Cove, NY, United States; ^2^Medical Scientist Training Program, Stony Brook University, Stony Brook, NY, United States; ^3^Department of Infection Control, Huntington Hospital, Northwell Health, Huntington, NY, United States; ^4^The Feinstein Institutes for Medical Research, Manhasset, NY, United States; ^5^Elmezzi Graduate School of Molecular Medicine, Manhasset, NY, United States; ^6^Department of Critical Care Medicine, Huntington Hospital, Northwell Health, Huntington, NY, United States; ^7^Department of Infectious Disease, Huntington Hospital, Northwell Health, Huntington, NY, United States

**Keywords:** *Corynebacterium diphtheriae*, *Corynebacterium diphtheriae* infection, non-toxigenic *Corynebacterium diphtheriae*, *C. diphtheria*, prolonged QT syndrome, critical care medicine

## Abstract

Here we document a rare, acute, infection caused by non-toxigenic *Corynebacterium diphtheriae* and the resulting unique and severe clinical sequelae. Our patient was a young man with no known pre-existing conditions that presented in cardiopulmonary arrest. We contrast this case with prior instances of non-toxigenic *C. diphtheriae* strain infection in the United States and summarize the literature that suggests systemic infection can result in cardiogenic toxicity. We speculate on a possible missed, pre-existing condition that could have increased this patient’s susceptibility to poor clinical outcome.

## Background

*Corynebacterium diphtheriae* is a gram-positive, rod-shaped, non-motile, non-sporulating, unencapsulated bacillus (1, 2). Infection with toxigenic strains is responsible for the well-known set of clinical symptoms that characterize the communicable disease-respiratory diphtheria. Respiratory diphtheria presents with fever, malaise, laryngeal swelling and occlusion followed by bacteremia and toxemia, and respiratory distress typically following the development of a pseudomembranous formation.

The introduction of toxoid-based, multi-dose, childhood Tetanus-Diphtheria-Pertussis vaccination (DTaP) and adult Tetanus-Diphtheria vaccination (Tdap) and booster (Td) largely eradicated cases of respiratory diphtheria in the United States (U.S.) and other developed countries (3). Since 2018, only a handful of cases have been reported in the U.S. each year, typically among vulnerable populations (4) including immigrants (5), the incarcerated (6), and individuals with vaccine hesitancy (7, 8). These socioeconomic variables are disproportionately distributed across individuals within a population, providing a substantial amount of complexity when it comes to population-level health policies and approaches to preventative healthcare and civilian education thereof (9, 10). In the U.S., population-level data estimate children aged 13–17 have 90.2% compliance with DTaP vaccines (3), a level considered sufficient to achieve herd immunity, but one that nevertheless leaves some potential for propagation and outbreak (11, 12). The relationship between population-level vaccine coverage and genomic mutation of *C. diphtheriae*, particularly relating to antigenic structures or pathogenic properties, is an area of ongoing investigation (13, 14).

Seroconversion following the primary series of Tdap vaccination in children is high (94% of patients), although this rate drops when vaccination occurs off-schedule (15). For adults without prior vaccination, the standard of care is to provide three doses of diphtheria-targeting vaccine including one Tdap (3, 16). Single doses of Tdap in immunocompetent, adult patients are reported to trigger sufficient seroconversion rates (17) and are not associated with greater incidence of infection (18), which suggests adult boosters may lack additional benefit. However, multiple doses in adults remains the gold standard for achieving long-lasting immunity. In the U.S., Td boosters are widely accessible, and their use is reimbursed by all major insurance companies, including the Medicare system.

However, vaccination does not protect directly against infection with non-toxigenic strains of *C. diphtheriae*. Toxigenic and non-toxigenic strains of *C. diphtheriae* are morphologically indistinguishable (1, 2). Non-toxigenic variants represent lower disease burden in terms of incidence of respiratory, disseminated infection, and clinical severity. Nevertheless, infection with non-toxigenic *C. diphtheriae* can result in endocarditis, osteomyelitis, and septic arthritis (19). Like toxigenic variants, disease resulting from infection with non-toxigenic *C. diphtheriae* is more common in vulnerable populations with pre-existing conditions (20). Non-toxigenic variants can also convert to toxigenic via bacteriophage-mediated lysogenesis (21). Taken together, the ability of non-toxigenic strains to acquire the *tox* gene puts vulnerable populations at risk for diphtheria outbreaks. Meanwhile, the prevalence of asymptomatic carriers of non-toxigenic strains is challenging to estimate and significant disease resulting from such strains is exceedingly rare within the U.S. (22–24).

Here we report one such case wherein infection with a non-toxigenic *C. diphtheriae* strain presented in a fully vaccinated young adult with no preexisting medical conditions. The challenges of this patient’s clinical presentations and management serve as important teaching points for critical care practitioners. Finally, this patient’s unique background provides important teaching points on the social determinants of health for preventative care medicine practitioners.

## Case report

### Initial presentation

**FIGURE 1:**
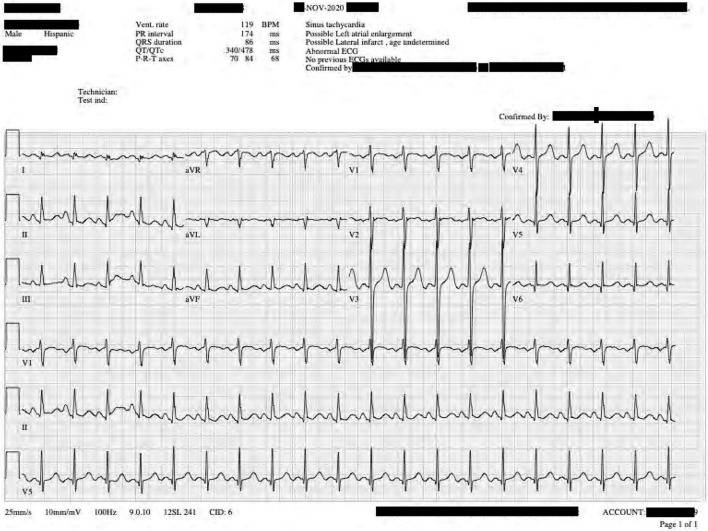
Twelve-lead electrocardiogram taken during the patient’s current presentation shortly after stabilization. T wave elevations are noted in leads II, V5–V6.

An 18-year-old adult, Hispanic male was presented to the Emergency Department (ED) by his family via personal automobile. According to the family, the patient was unconscious for approximately 10 minutes during which time he did not receive cardiopulmonary resuscitation (CPR). Upon arrival, he was found to be in cardiac arrest; the patient was not alert or oriented to person, place, or time, cool to the touch, had no agonal respirations, no radial pulse on palpation, no activity on cardiac monitor, no heartbeat on auscultation, pupils that were equal, and 4 mm dilated but non-reactive. The patient’s Glasgow Coma Scale score was 3. A CPR protocol was initiated immediately. Spontaneous circulation was achieved after three rounds of epinephrine administration. The patient was admitted to an isolated room in the Intensive Care Unit (ICU), intubated, and placed on a ventilator with a sedation regimen. A 12-lead EKG was obtained following stabilization of the patient demonstrating ST changes consistent with lateral wall ischemia (Figure 1).

#### Vitals and diagnostic work-up

Vital signs, laboratory values, including a toxicology screen, and urinalysis (Table 1) were all unremarkable; urine culture had no growth (Table 2). COVID-19 and Rapid Viral Panel testing, including for influenza were returned as negative. Preliminary treatment for sepsis and leukocytosis included intravenous 3.375 g combination piperacillin-tazobactam q.8.h. and 1,000 mg vancomycin q.d.

**TABLE 1:** Summary of vital signs and laboratory findings during current and prior presentation.

Order	Lab	Results (units) Current presentation	Results (units) Prior presentation
Vitals	Temperature	32.7^°^C	36.2^°^C
	Heart Rate/Pulse	37 bpm	54 bpm
	Blood Pressure	89/49 mmHg	92/51 mmHg
	Respiratory Rate	10 breaths/minute	16 breaths/minute
	O_2_ Saturation	80%	95%
CBC	WBC	11.88 Billion/L	11.80 Billion/L
	HB	13.7 mg/dL	12.4 mg/dL
	INR	1.27	1.33
Electrolyte panel	Na^+^	140 mEq/L	138 mEq/L
	K^+^	4.3 mEq/L	3.2 mEg/L
Other bloods	Glucose	584 mg/dL	456 mg/dL
	Lactate	3.4 mmol/L	12.8 mmol/L
Urinalysis	Bilirubin	Negative	Negative
	Urobilinogen	Negative	Negative
	Ketone	Negative	Negative
	Specific gravity	1.02	1.01
	Protein	100 mg/dL	30 mg/dL
	Nitrite	Negative	Negative
	Leukocyte esterase	Negative	Negative
	Blood	Trace	Trace
	Glucose	1,000 mg/dL	1,000 mg/dL
	pH	6.0	6.0
Other labs	COVID-19	Negative	Negative
	Blood cultures (see Table 2)	Positive*-Corynebacterium diphtheriae*	Positive*-Klebsiella*
		Positive*-Klebsiella pneumoniae*.	*pneumoniae*
	Urine toxicology	Acetaminophen-Negative	Acetaminophen-Negative
		Salicylate-Negative	Salicylate-Negative
		Benzodiazepines-Negative	Benzodiazepines-Negative
		THC-Negative	THC-Negative
		Cocaine-Negative	Cocaine-Negative
		Amphetamine-Negative	Amphetamine-Negative
		Barbiturates-Negative	Barbiturates-Negative
		Opiates-Negative	Opiates-Negative
		Phencyclidine-Negative	Phencyclidine-Negative
		Methadone- Negative	Methadone- Negative

Complete blood count (CBC), electrolyte panel, glucose, and lactate levels are provided as well as urine toxicology screen and COVID-19 test results. WBC, white blood cells; INR, international normalized ratio; HB, hemoglobin; Na^+^, serum sodium; K^+^, serum potassium; O_2_, oxygen; bpm, beats per minute.

**TABLE 2:** Blood, sputum, and urine culture results during prior and current visit.

Culture	Organism	Susceptibility results (mcg/L) Current presentation	Susceptibility results (mcg/L) Prior presentation
Blood	*Klebsiella pneumoniae*	Amikacin S ≤ 16	Amikacin S ≤ 16
		Ampicillin R > 16	Ampicillin R > 16
		Ampicillin/Sulbactam S ≤ 4/2	Ampicillin/Sulbactam S ≤ 4/2
		Aztreonam S ≤ 4	Aztreonam S ≤ 4
		Cefazolin S ≤ 2	Cefazolin S ≤ 2
		Cefepime S ≤ 2	Cefepime S ≤ 2
		Cefoxitin S ≤ 8	Cefoxitin S ≤ 8
		Ceftriaxone S ≤ 1	Ceftriaxone S ≤ 1
		Ciprofloxacin S ≤ 0.25	Ciprofloxacin S ≤ 0.25
		Ertapenem S ≤ 0.5	Ertapenem S ≤ 0.5
		Gentamicin S ≤ 2	Gentamicin S ≤ 2
		Imipenem S ≤ 1	Imipenem S ≤ 1
		Levofloxacin S ≤ 0.5	Levofloxacin S ≤ 0.5
		Meropenem S ≤ 1	Meropenem S ≤ 1
		Piperacillin/Tazobactam S ≤ 8	Piperacillin/Tazobactam S ≤ 8
		Tobramycin S ≤ 2	Tobramycin S ≤ 2
		Trimethoprim/Sulfamethoxazole S ≤ 0.5/9.5	Trimethoprim/Sulfamethoxazole S ≤ 0.5/9.5
	*Corynebacterium diphtheriae*	n/a	
Urine	No growth	n/a	n/a
Sputum	*Stenotrophomona maltophilia*	Ceftazidime | 16	
		Levofloxacin S ≤ 0.5	
		Trimethoprim/Sulfamethoxazole S ≤ 0.5/9.5	
	*Staphylococcus aureus*	Ampicillin/Sulbactam S ≤ 8/4	
		Cefazolin S ≤ 4	
		Clindamycin R < 0.25	
		Erythromycin R > 4	
		Gentamicin S ≤ 1	
		Oxacillin S ≤ 0.25	
		Penicillin R | 4	
		Rifampin S ≤ 1	
		Tetra/Doxy S ≤ 1	
		Trimethoprim/Sulfamethoxazole S ≤ 0.5/9.5	
		Vancomycin S | 1	

Corresponding sensitivity profiles-minimum inhibitory concentration (MIC)-based on multiplex PCR assay for 66 bacterial and resistance gene targets. R, resistant; S, sensitive; n/a, not applicable.

Blood samples from two access sites for culture and gram stain were collected. Draws from both sites grew *C. diphtheriae* (anaerobic bottles) and *Klebsiella pneumoniae* (both aerobic and anaerobic bottles) as identified with polymerase chain reaction (PCR) analysis (Table 2). The New York State Department of Health (NYDOH) and the U.S.’s Center for Disease Control’s (CDC) Emergency Operations Center were contacted promptly. Patient samples were shipped overnight for confirmatory testing. Per that NYDOH laboratory, again real time PCR amplified *C. diphtheriae* DNA, but not toxin A or toxin B DNA fragments.

The patient’s social and vaccination history was accertained. The patient was reported to have immigrated to the U.S. from Honduras 3 years prior at the age of 15. At the time of admission, he did not have citizenship, but had attended public high school, which requires by New York State Public Health law, documentation of his vaccination and medical history by a U.S.-based primary care physician. The patient’s family denied history of substance abuse disorders, smoking history, high risk sexual behavior, or recent travel. He was taking no medications and had no known complaints.

The patient’s primary care pediatrician was contacted who verified this medical and social history and confirmed full and timely vaccination status including for hepatitis A and B, human papillomavirus, meningococcal B, pneumococcal conjugate, varicella and influenza. Documentation was obtained from that source, and per those records he received 4 out of 5 recommended DTaP vaccinations- at ages 3 months, 5 months, 8 months, and 2 years—in Honduras, a country that successfully converted from a toxoid-based to DTaP vaccination system in 1998 as recommended by the World Health Organization and United Nations International Children’s Emergency Fund (25), based on the similarity of side-effect profiles, marginal cost increase, and more sustained immunity (26). Honduras has reported rates of DTaP vaccination comparable to the U.S. since 2009 (27). Further, the patient received a TDaP booster in the U.S. at age 15; therefore, by all accounts our patient had an up-to-date vaccination series.

### Prior presentation

Review of the patient’s medical charts revealed one other admission nearly 1 year prior to the current presentation. At that time, the patient also presented in a state of unresponsiveness after he was found reportedly “sleeping” in his room by his sibling who described labored breathing. The patient presented to the hospital in a state of shock. He received atropine 0.5 mg IV bolus and fluids. The patient was sedated, intubated, and transferred to the ICU. Naloxone (Narcan) 0.4 mg IV was administered empirically, and Psychiatry Services were consulted for possible toxin ingestion. Urine and blood toxicology analysis were unremarkable (Table 1) and urine culture had no growth (Table 2). COVID-19 PCR testing from nasal swab sample collection was negative. Initial, two-draw blood cultures grew *K. pneumoniae* after 36 h in one bottle, and a subsequent second draw 48 h later had no growth. The patient’s antibiotic treatment included four 1 g doses of third-generation cephalosporin (Ceftriaxone) b.i.d. for empiric, broad-spectrum coverage as recommended by the Infectious Disease team which also prescribed a 10-day course with 875/125 mg Amoxicillin/Clavulanate potassium (Augmentin) b.i.d. by mouth outpatient. As the patient could not be directly interviewed during his acute presentations we cannot be sure whether the patient was experiencing symptoms prior to his presentation. This is in part why the Pediatric Infectious Disease services empirically treated the patient for *K. pneumo* at the time of his first admission.

**FIGURE 2:**
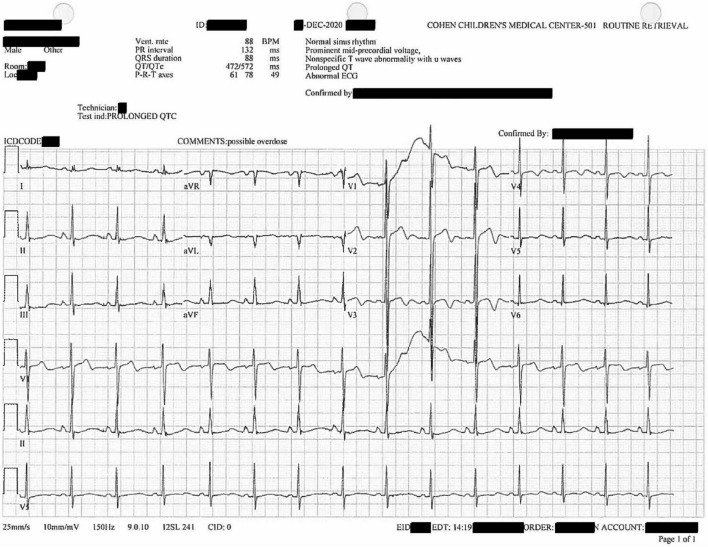
Twelve-lead diagnostic electrocardiogram collected after the patient’s initial hospitalization. U waves noted in V1–V4. Prolonged QTc of 572 ms.

**FIGURE 3:**
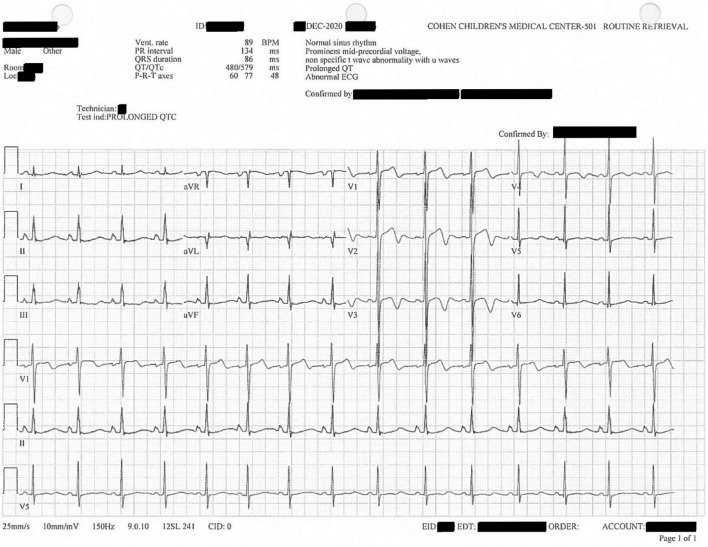
Confirmatory, diagnostic twelve-lead electrocardiogram replicating prior findings of V1–V4 U waves and prolonged QTc, here 579 ms.

Further workup included a computerized tomography scan of the cranium, cervical spine, abdomen, thorax; no significant findings were reported. Cardiac electrocardiography (ECG) showed sinus tachycardia and a prolonged QTc interval of 572 ms (Figure 2). Repeat cardiac ECG showed a QTc duration of 579 ms (Figure 3). No medications associated with medication-induced QT syndrome were given during his hospitalization. A limited cardiac echocardiogram study was performed during which coronary arteries were not visualized.

The patient was extubated and discharged 4 days following his admission. He was scheduled for outpatient follow up with Pediatric Cardiology Services. A stress test performed in that setting was normal with one premature ventricular contraction. Repeat cardiac echocardiogram and CT angiogram of coronary arteries were performed showing non-structural cardiac disease. He was referred for genetic testing for further work-up of a suspected genetic cardiac abnormality due to a family-reported (but unconfirmed) paternal uncle who passed away suddenly from a heart attack at the age of 40 in Honduras. The patient did not follow up for this genetic testing, likely due to financial limitations.

### Progression and resolution of the case

Presently, testing confirmed the strain to be non-toxigenic. As per the CDC’s Investigational New Drug protocol, antitoxin is only in cases of early detection of toxin-producing strains (3). As per official guidance (2), the patient was placed on droplet precaution isolation and contact-tracing of household members was conducted. All family members and close contacts were confirmed to be asymptomatic. Nasal swabs of four of the patient’s nine immediate household members came back positive for *C. diphtheriae*, indicating carrier status. In addition to Tdap boosters, IM Penicillin G for adult, family household and close contacts were chosen for treatment after discussion between Suffolk County Department of Health and the in house Infectious Disease team. The patient’s antibiotic course of piperacillin-tazobactam was extended, and cefepime 2,000 mg q.d. was added to the regime. Subsequent two-draw blood cultures on days 2 and 5 had no growth.

The patient’s progress was poor; due to the extended period without protected airway and circulation, the patient suffered irreversible anoxic brain injury. His family asked that “everything be done for him” and that the Chaplain’s Office, wherein representatives provide spiritual or religious support at the bedside, be involved in his case. Palliative Care Services were also consulted.

Given the patient’s prior cardiac history and workup, and current presentation of cardiac arrest with pulseless electrical activity, Electrophysiology (EP) Services was consulted, and the patient was kept on telemetry monitoring as part of his ICU care. That team confirmed the likelihood of a prolonged QTc and recommended continued avoidance of exacerbating medications for which review of concomitant medications was performed. A 2D echocardiogram demonstrated left ejection fraction (LVEF) of 50–55%. The patient was not considered a candidate for any EP intervention given his poor functional status and prognosis.

On day 2 of admission, the patient deteriorated into status epilepticus and was started on 1,000 mg IV levetiracetam q.12.h. Overnight on day 2, he was noted to have a fever and continued to spike fevers daily (maximum temperature of 39.0^°^C) for a period of 4 days. A suprapubic catheter and feeding tube were placed. On day 9 of the patient’s admission, his ventilatory status improved and a tracheostomy was performed, but he continued to produce oropharyngeal secretions requiring suctioning every few hours. Two bronchoscopies were performed; cultures of which grew ventilator-associated species- *Staphylococcus aureus* and *Stenotrophomonas maltophilia* (Table 2), for which he received 4 doses of 1,000 mg vancomycin q.d. and 12 doses of 160/800 mg trimethoprim-sulfamethoxazole q.12.h. Over 34 days he sustained a 20 lb weight loss, reaching a clinically underweight BMI of 16 despite supplementation with enteral nutrition. The patient’s condition ultimately stabilized; he was discharged to a long-term care facility 45 days following his admission as per his family’s wishes.

## Discussion

To the best of our knowledge this is the second case of systemic infection with a strain of *C. diphtheria* in the U.S. in 2021. Similar cases of bacteremia and significant clinical sequelae resulting from non-toxigenic diphtheria are rare (28). In developed countries, such cases are even rarer and typically present in patients with an immunocompromised condition, such as those with a history of IV drug use (29, 30). Our patient had no known pre-existing conditions (immunological or otherwise), was established with preventative, outpatient care, and had no incidental diagnosis despite prior hospitalization and work up by specialists. Cardiogenic toxicity due systemic infection with *C. diphtheria* is uncommon in developed countries (31). Such cases resulting from non-toxin producing strains are rare, even outside of the U.S. (30). These factors taken together make the case unusual. Further, such infections usually present as cases of endocarditis and/or thrombotic embolic events (31), not as cardiac arrest. Our patient has had multiple trans-thoracic echocardiograms and point of care ultrasound, which did not show any signs of endocarditis. To the best of our examination and work up, structurally, his heart was within normal ranges of efficiency and function.

We suspect that the patient held a congenital prolonged QT syndrome that remained subclinical up to the time of his first admission and that was possibly exacerbated by his infectious, inflammatory state as has been suggested by prior literature (32, 33). This is consistent with his family history and his prior presentation. Our patient underwent cardiac testing in the months prior to his second presentation. Largely that workup was insufficient for identifying etiologies that could explain his initial presentation in cardiac arrest to our hospital. However, congenital prolonged QT syndrome (LQTS) bestows propensity for cardiac arrest and can be challenging to diagnose properly (34). Our suspicion is further supported by reports in which infection in patients with established LQTS has triggered cardiologic de-stabilization, including infection with H1N1 influenza (35) and COVID-19 (36).

Finally, we acknowledge the cultural and socio-economic factors present in our patient that are associated with greater overall health risks and complications. Social and cultural determinants of health are a much-discussed issue which result in lack of access to, and use of, preventive and primary care, as well as lack of established trust with the medical system (37–39). Our patient was an immigrant American; the social “status” of the patient and his family is an important factor in the delay of the patient’s presentation to medical facilities when urgent care was needed. Further, the patient’s socioeconomic standing made genetic testing, potentially life-saving diagnostic information, financially unviable. These factors jeopardized optimal clinical outcomes for our patient in the context of preventative medical care, and of the imminent medical treatment he received after the onset of his illness.

The case characterized here resulted in a complex cascade of clinical consequences that we hope will spur conversation among critical care, infectious disease, medical genetic, and cardiology specialists. We hope practitioners will consider at which points in the healthcare system efforts could have ameliorated a better outcome and the lingering future threat of disease resulting from infection with non-toxigenic forms of *C. diphtheriae* in vaccinated populations.

## Data availability statement

The original data contributions presented in this study are included in the article/supplementary material, further inquiries can be directed to the corresponding author.

## Ethics statement

Ethical review and approval was not required for the study on human participants in accordance with the local legislation and institutional requirements. Written informed consent from the patient or next of kin was not required to participate in this study in accordance with the national legislation and the institutional requirements.

## Author contributions

GA and DR were responsible for manuscript drafting and editing. DR was responsible for literature review, final formatting, and submission. SS facilitated discussions with the CDC and NYS Department of Health. GA, SS, SM, SG, PS, and CH oversaw care of the patient, interpreted patient data, and contributed meaningful discussions and edits to the manuscript. All authors approved the final version of the work.
